# Association Between HIV-1 RNA Detection Status and HBV DNA Detection: A Patient-Level Retrospective Longitudinal Molecular Study from Istanbul, Türkiye

**DOI:** 10.3390/diagnostics16193214

**Published:** 2026-10-04

**Authors:** Murat Yaman, Kubra Alay Kara

**Affiliations:** 1 Medical Microbiology, Marmara University Pendik Research and Training Hospital, Istanbul 34899, Turkey; 2 Medical Microbiology, Kartal Dr. Lutfi Kirdar City Hospital, Istanbul 34865, Turkey; kubraalaykara@gmail.com

**Keywords:** HIV-1, hepatitis B virus, HBV DNA, HIV–HBV coinfection, molecular detection, longitudinal monitoring, real-time PCR

## Abstract

**Background/Objectives**: HIV and hepatitis B virus (HBV) share major transmission routes and may coexist in the same individual, making coordinated molecular assessment clinically important. **Methods**: This retrospective longitudinal laboratory-based study evaluated the patient-level association between HIV-1 RNA detection and HBV DNA detection using routine molecular testing data from a tertiary-care hospital in Istanbul, Türkiye. HIV-1 RNA and HBV DNA results obtained between 1 January 2022 and 1 August 2026 were linked at the patient level. A total of 5303 unique patients had HIV-1 RNA testing and 30,981 unique patient records had HBV DNA testing; these were separate source populations, and 529 individuals with results available for both molecular assays constituted the primary analytical cohort. **Results**: Among the 529 patients, HIV-1 RNA was detected at least once in 246 (46.5%), while HBV DNA was detected at least once in 147 (27.8%). HBV DNA detection occurred in 83/246 (33.7%) patients with at least one detectable HIV-1 RNA result and in 64/283 (22.6%) patients with no detectable HIV-1 RNA in any available measurement (OR, 1.74; 95% CI, 1.19–2.56; *p* = 0.005). However, the association was attenuated after adjustment for age and sex and was no longer statistically significant (adjusted OR, 1.28; 95% CI, 0.84–1.94; *p* = 0.255). HIV-1 RNA magnitude and HIV-1 RNA categories were not significantly associated with HBV DNA detection, and no significant correlation was observed between same-day HIV-1 RNA and HBV DNA levels (Spearman’s ρ = 0.161; *p* = 0.387) or between the nearest quantitative measurements obtained within ±30 days (ρ = 0.171; *p* = 0.245). Because treatment exposure and adherence information were unavailable, the observed molecular patterns could not be interpreted as direct measures of treatment response or treatment failure. **Conclusions**: These findings indicate that HIV-1 RNA detection status or magnitude should not be used as a surrogate for HBV DNA detection and support independent, clinically indicated HBV assessment in people living with HIV.

## 1. Introduction

Human immunodeficiency virus (HIV) and hepatitis B virus (HBV) infections remain major global public health challenges. According to the World Health Organization (WHO), approximately 41 million people were living with HIV worldwide at the end of 2025 [1]. In parallel, an estimated 240 million people were living with chronic HBV infection in 2024, with approximately 1.1 million deaths attributed to HBV-related cirrhosis and hepatocellular carcinoma [2]. Because HIV and HBV share major routes of transmission, particularly sexual and blood-borne exposure, their coexistence represents an important clinical and public health concern. The prevalence of HIV/HBV coinfection varies substantially according to geographical region, population characteristics, risk exposures, and HBV vaccination coverage [3].

HIV/HBV coinfection has been associated with increased HBV replication, impaired HBV clearance, progressive liver disease, and increased liver-related morbidity and mortality [4,5]. Accordingly, current guidelines recommend systematic assessment of HBV status in people living with HIV using hepatitis B surface antigen (HBsAg), total hepatitis B core antibody (anti-HBc), and hepatitis B surface antibody (anti-HBs). Quantitative HBV DNA assessment is recommended when HBV infection is identified, and antiretroviral therapy (ART) should be selected to provide activity against both viruses [6,7,8]. In particular, tenofovir alafenamide (TAF) or tenofovir disoproxil fumarate (TDF), in combination with emtricitabine (FTC) or lamivudine (3TC), is recommended as part of a fully suppressive ART regimen in individuals with HIV/HBV coinfection. Vaccination of non-immune individuals and confirmation of post-vaccination serological response are also particularly important in people living with HIV [6,7,8].

However, virological control of HIV and HBV does not necessarily occur in parallel. Previous studies of HIV/HBV-coinfected cohorts receiving tenofovir-based therapy have suggested that detectable HIV RNA may be associated with delayed or incomplete HBV DNA suppression. Conversely, persistent HBV DNA detection has also been documented in some patients despite sustained HIV RNA suppression [9,10,11,12]. These observations indicate that the relationship between HIV and HBV molecular activity is complex and may not be adequately explained by HIV-1 RNA detection or magnitude alone.

Studies from Türkiye have provided important data regarding HBV seroprevalence, occult HBV infection, isolated anti-HBc positivity, and responses to HBV-active ART among people living with HIV [13,14,15,16,17]. Nevertheless, data evaluating HIV-1 RNA detection status, HBV DNA detection, and quantitative viral loads simultaneously at the patient level within large routine molecular laboratory datasets remain limited.

Therefore, the primary aim of this study was to evaluate the patient-level association between HIV-1 RNA detection status and HBV DNA detection using longitudinal routine molecular testing data. Secondary objectives were to investigate the relationships of age, sex, and quantitative HIV-1 RNA and HBV DNA levels with these molecular patterns. We hypothesised that although HBV DNA detection may occur more frequently among patients with HIV-1 RNA detected during follow-up, the magnitude of HIV-1 RNA may not necessarily correlate directly with HBV DNA level, reflecting the distinct virological and therapeutic determinants governing the two infections.

## 2. Materials and Methods

### 2.1. Study Design and Data Source

This retrospective, laboratory-based longitudinal study included HIV-1 RNA and HBV DNA tests performed as part of routine clinical diagnosis and follow-up at the ISLAB_1 Central Laboratory of Kartal Dr. Lütfi Kırdar City Hospital, Istanbul, Türkiye, between 1 January 2022 and 1 August 2026.

Age, sex, patient identifiers, specimen/test dates, and molecular test results were retrieved from the Laboratory Information Management System (LIMS). Longitudinal follow-up tests performed on different dates for the same patient were retained, whereas true duplicate reports of the same test were excluded.

Patient-level record linkage prioritised available unique identifiers. When unique identifiers were unavailable, normalised first name and surname, sex, and age were jointly evaluated for record matching. Records containing conflicting identification information were not automatically merged. Only patients with confirmed results for both molecular tests were included in the primary patient-level analysis.

### 2.2. Molecular Testing

HIV-1 RNA and HBV DNA quantification were performed using the ELITe BeGenius^®^ platform (ELITechGroup SpA, Turin, Italy). HIV-1 RNA was measured using the HIV1 ELITe MGB^®^ Kit (ELITechGroup SpA, Turin, Italy) based on real-time reverse-transcription PCR, whereas HBV DNA was measured using the HBV ELITe MGB^®^ Kit (ELITechGroup SpA, Turin, Italy) based on real-time PCR. According to the manufacturer’s specifications for the ELITe BeGenius (ELITechGroup SpA, Turin, Italy) configuration, the analytical limit of detection (LoD) and lower limit of quantification (LLoQ) were 26 copies/mL (60 IU/mL) for HIV-1 RNA and 38 copies/mL (9 IU/mL) for HBV DNA, respectively. These analytical limits were used to interpret detected-but-not-quantifiable results.

Quantitative HIV-1 RNA results were expressed as copies/mL, whereas HBV DNA results were expressed as IU/mL. Results reported as below the lower limit of quantification but indicating target detection (“<” results) were classified as positive for binary detection analyses. Because the exact viral concentration was not available, these results were excluded from quantitative viral load analyses. Similarly, results exceeding the upper limit of quantification (“>” results) were excluded from analyses requiring exact quantitative viral load values. Because molecular assays cannot reliably exclude nucleic acid concentrations below their analytical detection limits, an undetected result should be interpreted as below the assay’s detection capability rather than as definitive absence of viral nucleic acid.

### 2.3. Study Outcomes and Variables

The primary outcome was the detection of HBV DNA at least once during the study period. Patients were classified according to their longitudinal HIV-1 RNA status as those with HIV-1 RNA detected at least once during the study period and those in whom HIV-1 RNA was not detected in any available measurement. For secondary analyses, age was categorised as <30, 30–39, 40–49, and ≥50 years. For quantitative analyses, the maximum exact quantifiable HIV-1 RNA or HBV DNA value recorded for each patient during the study period was used.

HIV-1 RNA results were additionally categorised as detected below the limit of quantification, <200, 200–999, and ≥1000 copies/mL. The relationship between concurrent molecular levels was assessed using HIV-1 RNA and HBV DNA measurements obtained on the same day. As a sensitivity analysis, the closest pair of quantitative results obtained within ±30 days was also evaluated. These temporal analyses incorporated limited within-patient timing information but did not constitute a full longitudinal repeated-measures model.

Thus, although all available longitudinal measurements were retained in the dataset, the primary association analysis was an occurrence-based patient-level comparison (ever detected versus never detected) rather than a formal repeated-measures model.

### 2.4. Ethical Approval

This retrospective study was approved by the Clinical Research Ethics Committee of Kartal Dr. Lütfi Kirdar City Hospital Scientific Research Ethics Committee Decision Form (protocol code 2026/0l0.99/3 l/55, date of approval 26.08.2026). All data were processed in anonymised form. The requirement for individual informed consent was waived because the study used retrospective, anonymised routine-care laboratory data and did not include directly identifiable personal information.

### 2.5. Statistical Analysis

All retrospective data retrieved from the LIMS were recorded in Microsoft Excel, version 16 (Microsoft Corp., Redmond, WA, USA). Statistical analyses were performed using IBM SPSS Statistics version 27.0 (IBM Corp., Armonk, NY, USA) and GraphPad Prism version 10.0 (GraphPad Software, San Diego, CA, USA).

Categorical variables were summarised as numbers and percentages, whereas continuous variables were summarised as medians and interquartile ranges (IQRs). Pearson’s chi-square test or Fisher’s exact test was used for comparisons of categorical variables. The Mann–Whitney U test or Kruskal–Wallis test was used for comparisons of continuous variables, as appropriate.

For the primary comparison, odds ratios (ORs) and corresponding 95% confidence intervals (CIs) were calculated. Relative risks (RRs) were additionally estimated where appropriate. A multivariable logistic regression model including HIV-1 RNA detection status, sex, and age group was constructed to evaluate factors associated with HBV DNA detection. Associations between quantitative HIV-1 RNA and HBV DNA levels were assessed using Spearman’s rank correlation coefficient.

All statistical tests were two-sided, and a *p*-value < 0.05 was considered statistically significant.

### 2.6. Data Availability

The data supporting the findings of this study were derived from routine clinical laboratory records and contain potentially identifiable patient information. Therefore, the underlying individual-level dataset is not publicly available. De-identified data may be made available by the corresponding author upon reasonable request and subject to institutional and ethical approval.

### 2.7. Generative Artificial Intelligence Disclosure

Generative artificial intelligence tools were used for language editing support. All decisions regarding study design, data collection, statistical analyses, interpretation of results, and final scientific content were reviewed and approved by the authors; the authors are fully responsible for the accuracy and integrity of the article.

## 3. Results

### 3.1. Overview of Molecular Testing and Patient-Level Cohort

During the study period, a total of 23,966 HIV-1 RNA results from 5303 unique patients were evaluated, and HIV-1 RNA was detected at least once in 2611 patients (49.2%). The median number of HIV-1 RNA tests per patient was 3 (IQR, 1–7; range, 1–47). During the same period, 75,792 HBV DNA results from 30,981 unique patient records were evaluated, and HBV DNA was detected at least once in 13,948 records (45.0%). The median number of HBV DNA tests per unique record was 1 (IQR, 1–3; range, 1–25). The 5303 HIV-1 RNA-tested patients and 30,981 HBV DNA-tested records represented separate source populations rather than one being a subset of the other. Patient-level linkage identified 529 individuals with results available for both molecular assays (Figure 1A).

Following patient-level cross-matching, 529 unique patients with results available for both molecular tests were included in the primary analysis. Among these patients, HIV-1 RNA was detected at least once in 246 (46.5%), whereas HIV-1 RNA was not detected in any available measurement in 283 (53.5%). HBV DNA was detected at least once in 147 of 529 patients (27.8%) (Table 1). The matched cohort comprised four mutually exclusive molecular detection patterns: HIV-1 RNA detected/HBV DNA detected in 83 patients, HIV-1 RNA detected/HBV DNA not detected in 163, HIV-1 RNA not detected/HBV DNA detected in 64, and HIV-1 RNA not detected/HBV DNA not detected in 219. These molecular categories do not establish previous exposure, chronic infection, or serological status in the absence of corresponding clinical and serological data.

### 3.2. Association Between HIV-1 RNA Status and HBV DNA Detection

HBV DNA was detected in 33.7% (83/246) of patients with HIV-1 RNA detected at least once, compared with 22.6% (64/283) of patients in whom HIV-1 RNA was not detected in any available measurement. This difference was statistically significant (OR, 1.74; 95% CI, 1.19–2.56; RR, 1.49; 95% CI, 1.13–1.97; *p* = 0.005) (Figure 1B and Table 2).

HBV DNA was detected in 33.7% (83/246) of patients with HIV-1 RNA detected at least once, compared with 22.6% (64/283) of patients in whom HIV-1 RNA was not detected in any available measurement. This difference was statistically significant in the unadjusted analysis (OR, 1.74; 95% CI, 1.19–2.56; RR, 1.49; 95% CI, 1.13–1.97; *p* = 0.005) (Figure 1B and Table 2). However, after adjustment for age and sex, the association was attenuated and was no longer statistically significant (aOR, 1.28; 95% CI, 0.84–1.94; *p* = 0.255) (Figure 1C and Table 3 and Table 4).

### 3.3. Associations of Age and Sex with HBV DNA Detection

HBV DNA detection rates differed significantly across age groups, increasing from 8.2% among patients aged <30 years to 32.7% among those aged 30–39 years, peaking at 35.9% among those aged 40–49 years, and decreasing to 28.6% among patients aged ≥50 years (*p* < 0.001). HBV DNA was also detected more frequently in males than in females (30.4% vs. 19.8%, respectively; *p* = 0.024) (Table 4).

In the multivariable logistic regression model, age remained independently associated with HBV DNA detection. Using patients aged <30 years as the reference group, the adjusted odds ratios were 4.09 (95% CI, 1.75–9.57; *p* = 0.001) for those aged 30–39 years, 6.09 (95% CI, 2.65–13.98; *p* < 0.001) for those aged 40–49 years, and 4.17 (95% CI, 1.90–9.15; *p* < 0.001) for those aged ≥50 years. In contrast, HIV-1 RNA detection at least once (aOR, 1.28; 95% CI, 0.84–1.94; *p* = 0.255) and male sex (aOR, 1.56; 95% CI, 0.94–2.60; *p* = 0.085) were not statistically significant after adjustment (Figure 1C and Table 3).

### 3.4. Quantitative Viral Load Analyses

Among patients with exact quantitative results, the maximum HIV-1 RNA level was similar between patients with and without HBV DNA detected (*n* = 79; median, 87,138 copies/mL; IQR, 8400–338,806) and those without HBV DNA detected (*n* = 152; median, 83,813 copies/mL; IQR, 10,061–372,333; *p* = 0.994). HBV DNA detection rates also did not differ significantly across HIV-1 RNA categories (*p* = 0.775) (Table 5). These findings did not demonstrate an association between HIV-1 RNA magnitude and HBV DNA detection.

Similarly, the maximum HBV DNA level did not differ significantly between patients with HIV-1 RNA detected at least once (*n* = 75; median, 5350 IU/mL; IQR, 318–1,508,454) and those with no HIV-1 RNA detected in any available measurement (*n* = 56; median, 2117 IU/mL; IQR, 96–14,048; *p* = 0.106).

Among 31 patients with quantitative HIV-1 RNA and HBV DNA results obtained on the same day, no significant correlation was observed between the two viral loads (Spearman’s ρ = 0.161; *p* = 0.387). Similarly, analysis of the nearest quantitative HIV-1 RNA and HBV DNA measurements obtained within ±30 days showed no significant correlation (*n* = 48; Spearman’s ρ = 0.171; *p* = 0.245) (Table 5). These paired analyses provide limited temporal information but do not constitute a formal repeated-measures analysis.

## 4. Discussion

In this patient-level analysis, HBV DNA detection was more frequent among patients with at least one detectable HIV-1 RNA result than among those with no detectable HIV-1 RNA throughout the study period. Although this difference was statistically significant in the unadjusted analysis, the association was attenuated after adjustment for age and sex and was no longer statistically significant. Thus, the unadjusted association should not be interpreted as evidence of an independent relationship between HIV-1 RNA detection and HBV DNA detection. In contrast, age, particularly the 40–49-year group, showed the strongest independent association with HBV DNA detection. These findings indicate that demographic characteristics and clinical monitoring patterns may contribute to the observed molecular detection patterns.

An additional consideration is the difference in longitudinal testing intensity between the two molecular assays. The median number of HIV-1 RNA measurements was 3 per patient (IQR, 1–7; range, 1–47), whereas the median number of HBV DNA measurements was 1 per patient record (IQR, 1–3; range, 1–25). This discrepancy is consistent with the different clinical roles of the two assays. HIV-1 RNA quantification is routinely used for longitudinal monitoring of antiretroviral treatment response and maintenance of virological suppression and is therefore performed repeatedly during HIV care. In contrast, HBV DNA testing is generally guided by HBV serological status, treatment indication, and specific clinical circumstances. Accordingly, unequal observation intensity may have influenced the probability of identifying at least one positive result during the study period. This ascertainment effect is particularly relevant because the primary analysis reduced repeated measurements to patient-level ever-detected versus never-detected categories.

Previous studies in HIV/HBV-coinfected populations have suggested that detectable HIV viraemia may be associated with delayed or incomplete HBV DNA suppression. Matthews et al. reported an association between suboptimal adherence, detectable HIV RNA, and persistent HBV replication among individuals receiving tenofovir-based ART, while Hafkin et al. similarly found that detectable HIV viraemia was associated with incomplete HBV suppression during tenofovir therapy [9,10]. Longer-term studies have also shown that persistent or intermittent HBV DNA detection may occur in a subset of patients despite tenofovir-containing ART [18,19,20,21,22,23,24]. Together, these observations indicate that molecular responses to HIV and HBV may differ substantially between individuals and over time. However, treatment-related explanations cannot be directly evaluated in the present dataset because individual ART exposure and adherence were unavailable.

A particularly relevant finding of the present study was that HIV-1 RNA was not detected in any available measurement during the study period in 43.5% of patients in whom HBV DNA was detected. This finding indicates that the absence of detectable HIV-1 RNA during available measurements does not establish the absence of HBV DNA detection. Importantly, detectable HIV-1 RNA in a routine laboratory cohort may reflect several clinically distinct circumstances, including treatment-naïve or newly diagnosed infection, treatment interruption, incomplete adherence, treatment modification, or incomplete virological suppression. Similarly, HBV DNA detection cannot be interpreted as treatment failure without information on HBV-active treatment, treatment duration, adherence, HBsAg and HBeAg status, and HBV disease phase. These variables, together with CD4 cell counts and liver-related clinical parameters, were unavailable in the present dataset. According to the 2023 consensus report of the Turkish Society of Clinical Microbiology and Infectious Diseases, patients with confirmed HIV/HBV coinfection should generally receive a fully suppressive ART regimen containing tenofovir (TDF or TAF) together with an HBV-active NRTI such as FTC or 3TC, unless contraindicated [25]. Because treatment exposure was not available in this study, the molecular patterns observed here should not be interpreted as direct measures of treatment response or treatment failure.

The quantitative analyses further suggest that HIV-1 RNA magnitude and HBV DNA detection do not necessarily follow parallel patterns. Maximum HIV-1 RNA levels were similar in patients with and without HBV DNA detection, and HBV DNA detection rates did not differ significantly across HIV-1 RNA categories. Moreover, no significant correlation was observed between HIV-1 RNA and HBV DNA levels among same-day measurements or when the nearest quantitative test pairs within ±30 days were analysed. Therefore, our findings do not support a simple linear relationship in which increasing HIV-1 RNA is accompanied by proportionally increasing HBV DNA.

Nevertheless, the absence of a direct correlation between the two molecular levels should not be interpreted as indicating that HBV DNA lacks clinical importance. Serum HBV DNA levels have been associated with a biological gradient in the risk of HBV-related hepatocellular carcinoma [26]. In HIV/HBV coinfection, HIV and HBV molecular markers may also contribute to assessment of liver fibrosis and other adverse clinical outcomes [27], while persistent HBV replication has been associated with unfavourable long-term outcomes [28]. Thus, the clinical significance of HBV DNA should be interpreted independently of whether its concentration correlates directly with contemporaneous HIV viraemia.

The strong association between age and HBV DNA detection is also clinically and epidemiologically relevant. Zerdali et al. reported that HBV markers were more frequent among older people living with HIV in Istanbul [13], while Koçak et al. found an association between age and isolated anti-HBc positivity [15]. Surveillance data from the European Centre for Disease Prevention and Control have similarly shown that the highest notification rates for both acute and chronic hepatitis B were observed among adults aged 35–44 years, providing a broader epidemiological context for the age distribution observed in our cohort [29]. 

These observations support consideration of age together with HBV serological status, treatment history, and markers of liver disease when interpreting molecular detection patterns.

Studies from Türkiye have highlighted several aspects of HBV infection relevant to HIV care. A multicentre study identified occult HBV infection among HBsAg-negative people living with HIV [14], while studies from Istanbul have emphasised the importance of systematic evaluation of HBV serological status, vaccination history, and isolated anti-HBc patterns [13,15]. Multicentre treatment data from Türkiye have suggested high rates of HBV DNA suppression with TAF/TDF-containing regimens in HIV/HBV coinfection [16], and recent data from Ankara have further underscored the importance of monitoring viral hepatitis coinfections and viral load characteristics among people living with HIV [17]. These findings further support the need to interpret HBV DNA together with serological and treatment information rather than as an isolated molecular endpoint.

Importantly, the 27.8% rate of HBV DNA detection observed in the present matched cohort should not be interpreted as the prevalence of HIV/HBV coinfection. HBV DNA testing was not performed systematically in all individuals undergoing HIV-1 RNA testing and was likely concentrated among patients with known HBV infection, positive serological findings, treatment monitoring requirements, or clinical suspicion. Therefore, the study population represents a selected testing cohort rather than an unselected HIV population. The absence of comprehensive serological data also prevents definitive classification of molecularly defined groups as confirmed coinfection, previous exposure, or chronic HBV infection.

From a clinical perspective, our findings support the need to evaluate HBV independently of HIV-1 RNA status in people living with HIV. Current EASL, NIH, AASLD/IDSA, and Turkish consensus recommendations emphasise assessment of HBV serological markers, quantitative HBV DNA evaluation when indicated, vaccination of susceptible individuals, and selection of ART regimens that maintain activity against HBV [6,7,8,25]. Accordingly, systematic assessment of HBsAg, total anti-HBc, and anti-HBs; vaccination of non-immune individuals with confirmation of post-vaccination anti-HBs response when indicated; HBV DNA monitoring in HBsAg-positive or otherwise clinically appropriate patients; and coordinated continuation of HBV-active ART represent important components of integrated HIV/HBV care.

### Limitations

Several limitations should be considered. First, the retrospective laboratory-based design prevented integration of important clinical variables, including ART initiation and regimen, tenofovir exposure, treatment duration and adherence, CD4 cell count, HBeAg status, HBsAg status, HBV disease phase, and liver-related clinical parameters. Therefore, detectable HIV-1 RNA or HBV DNA could not be attributed to specific treatment-related or biological mechanisms. Second, although repeated measurements were retained, the primary analysis classified patients according to whether HIV-1 RNA or HBV DNA was detected at least once during the observation period; thus, the analysis did not fully exploit repeated-measures and temporal information and was not a formal repeated-measures model. Third, HIV-1 RNA and HBV DNA were not measured with equal frequency, which may have introduced differential ascertainment of molecular detection. Fourth, the analytical sensitivity of the assays limits the ability to identify nucleic-acid levels below the relevant detection capability; undetected results therefore cannot exclude concentrations below the assay LoD. Fifth, the matched cohort was derived from routine clinical testing and therefore represents a selected testing population rather than a population-based sample. Finally, the absence of comprehensive serological and clinical data precluded definitive classification of HIV/HBV coinfection, previous exposure, treatment status, or disease phase.

## 5. Conclusions

In this patient-level retrospective laboratory study, HBV DNA was detected more frequently among patients with at least one detectable HIV-1 RNA result than among those with no detectable HIV-1 RNA in the unadjusted analysis. However, this association was attenuated after adjustment for age and sex and was no longer statistically significant. HIV-1 RNA magnitude and HIV-1 RNA categories were not associated with HBV DNA detection, and no significant correlation was observed between contemporaneous HIV-1 RNA and HBV DNA levels.

These findings do not support the use of HIV-1 RNA detection or magnitude as a surrogate marker for HBV DNA detection. Interpretation of molecular findings should consider the unequal frequency of longitudinal testing and the fact that the primary analysis was based on patient-level ever-detected versus never-detected categories rather than a formal repeated-measures model. The four molecular detection patterns identified in the matched cohort should not be interpreted as evidence of previous exposure or confirmed coinfection without corresponding serological and clinical data.

Because ART exposure, HBV-active treatment, treatment duration, and adherence information were unavailable, the observed molecular patterns cannot be interpreted as direct measures of treatment response or treatment failure. In confirmed HIV/HBV coinfection, guideline-concordant care requires an ART regimen with activity against both viruses, while HBV assessment should be interpreted in conjunction with serology, clinical context, and treatment history.

Overall, the findings support continued independent assessment of HBV in people living with HIV rather than using HIV-1 RNA status as a surrogate for HBV molecular activity.

## Figures and Tables

**Figure 1:**
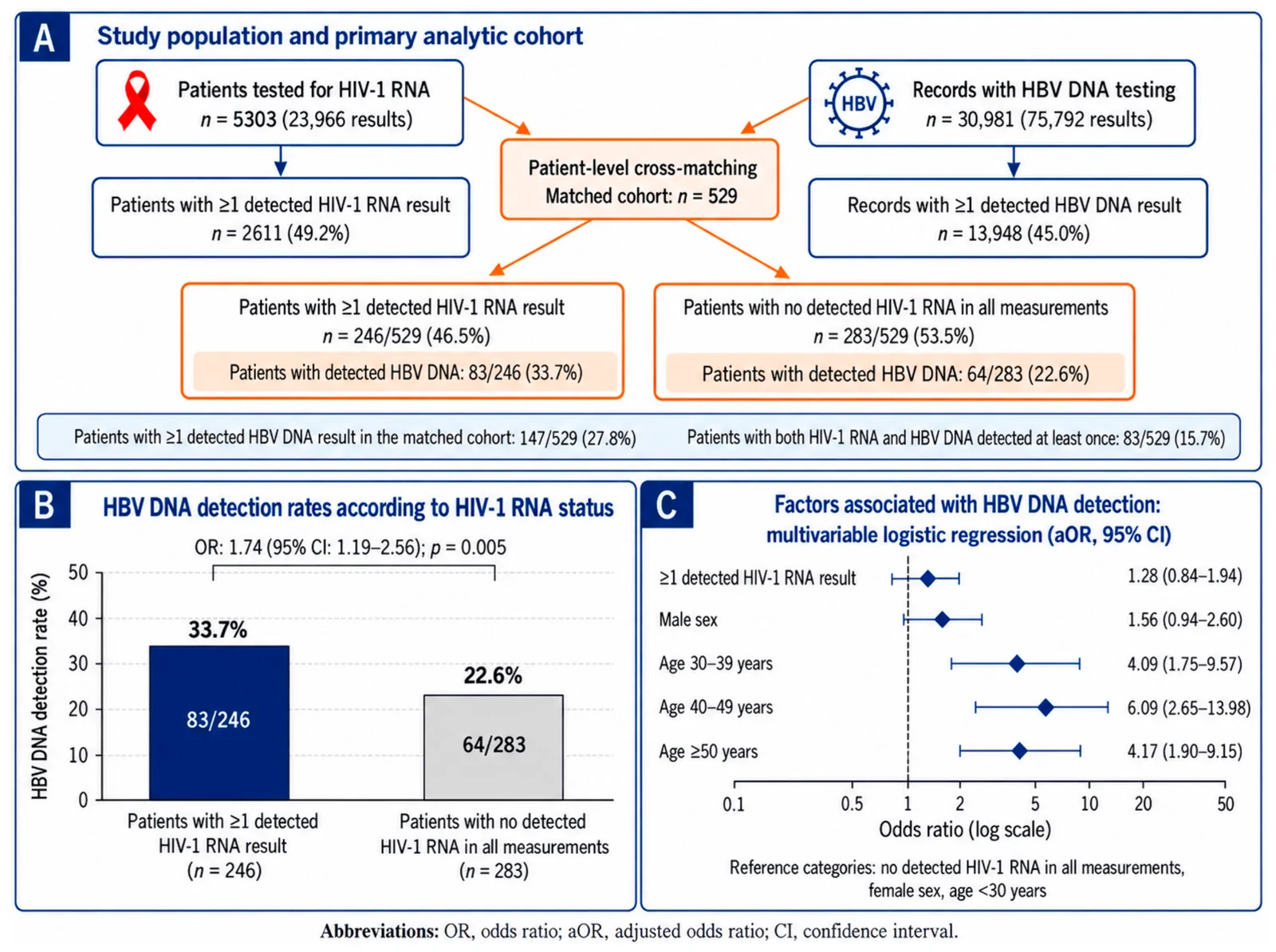
Patient-level molecular testing and analytical cohort. (**A**) Flow of patients from the HIV-1 RNA and HBV DNA source datasets to the matched analytical cohort. The two source populations were cross-matched at the patient level to identify individuals with results for both molecular assays. Among 529 matched patients, four mutually exclusive molecular detection categories were identified: HIV-1 RNA detected/HBV DNA detected (*n* = 83); HIV-1 RNA detected/HBV DNA not detected (*n* = 163); HIV-1 RNA not detected/HBV DNA detected (*n* = 64); and HIV-1 RNA not detected/HBV DNA not detected (*n* = 219). Molecular detection alone does not establish previous exposure or serological status. (**B**) HBV DNA detection rates according to HIV-1 RNA detection status in the matched cohort. (**C**) Multivariable logistic regression analysis of factors associated with HBV DNA detection. Reference categories were no detected HIV-1 RNA in any available measurement, female sex, and age <30 years. Abbreviations: OR, odds ratio; aOR, adjusted odds ratio; CI, confidence interval.

**Table 1:** Distribution of HIV-1 RNA and HBV DNA detection status in the patient-level study cohort.

Variable	n/N	%
Patients with both molecular tests	529	100
HIV-1 RNA detected at least once	246/529	46.5
No HIV-1 RNA detected in any available measurement	283/529	53.5
HBV DNA detected at least once	147/529	27.8
Both HIV-1 RNA and HBV DNA detected at least once	83/529	15.7

**Table 2:** Association between HIV-1 RNA detection status and HBV DNA detection at the patient level.

HIV-1 RNA Status	HBV DNA Detected, *n*	HBV DNA Not Detected, *n*	Total	HBV DNA Detection Rate (%)
HIV-1 RNA detected at least once	83	163	246	33.7
No HIV-1 RNA detected in any available measurement	64	219	283	22.6
Statistical comparison	OR: 1.74; 95% CI: 1.19–2.56	RR: 1.49; 95% CI: 1.13–1.97	*p* = 0.005	

**Table 3:** Factors independently associated with HBV DNA detection: multivariable logistic regression.

Variable	Adjusted OR (95% CI)	*p*-Value
HIV-1 RNA detected at least once	1.28 (0.84–1.94)	0.255
No HIV-1 RNA detected	Reference	—
Male	1.56 (0.94–2.60)	0.085
Female	Reference	—
<30 years	Reference	—
30–39 years	4.09 (1.75–9.57)	0.001
40–49 years	6.09 (2.65–13.98)	<0.001
≥50 years	4.17 (1.90–9.15)	<0.001

**Table 4:** Association of age group and sex with HBV DNA.

Variable	Male	Female	Total	HBV DNA Detected, *n* (%)	HBV DNA Not Detected, *n* (%)	*p*-Value
Age group						<0.001
<30 years	63	22	85	7 (8.2)	78 (91.8)	
30–39 years	84	26	110	36 (32.7)	74 (67.2)	
40–49 years	92	25	117	42 (35.9)	75 (64.1)	
≥50 years	159	58	217	62 (28.6)	155 (71.4)	
Sex						0.024
Male	398	131	529	121 (30.4)	277 (69.6)	
Female	-			26 (19.8)	105 (80.2)	

**Table 5:** Quantitative analyses of HIV-1 RNA and HBV DNA levels.

Analysis/HIV-1 RNA Category	*n*	HBV DNA Detected, *n* (%)	Result	*p*-Value
HIV-1 RNA viral-load categories				0.775
Detected below LLoQ	15	4 (26.7%)	—	
<200 copies/mL	25	6 (24.0%)	—	
200–999 copies/mL	16	5 (31.2%)	—	
≥1000 copies/mL	190	68 (35.8%)	—	
Between-group quantitative comparisons				
Maximum HIV-1 RNA in patients with HBV DNA detected	79	—	87,138 [8400–338,806] copies/mL	0.994
Maximum HIV-1 RNA in patients with HBV DNA not detected	152	—	83,813 [10,061–372,333] copies/mL	
Maximum HBV DNA in patients with HIV-1 RNA detected at least once	75	—	5350 [318–1,508,454] IU/mL	0.106
Maximum HBV DNA in patients with no HIV-1 RNA detected in any available measurement	56	—	2117 [96–14,048] IU/mL	
Correlation analyses				
Same-day HIV-1 RNA/HBV DNA correlation	31	—	Spearman’s ρ = 0.161	0.387
Nearest HIV-1 RNA/HBV DNA measurements within ±30 days	48	—	Spearman’s ρ = 0.171	0.245

Notes: For between-group quantitative comparisons, *n* represents patients with an exact quantifiable viral-load result contributing to the patient-level maximum; results reported only below the lower limit of quantification were not treated as exact quantitative values. The *p*-value of 0.775 refers to the comparison of HBV DNA detection across HIV-1 RNA viral-load categories.

## Data Availability

The data presented in this study are not publicly available due to ethical and privacy restrictions related to patient-level clinical laboratory data.
